# Reaching health facilities in situations of emergency: qualitative study capturing experiences of pregnant women in Africa’s largest megacity

**DOI:** 10.1186/s12978-020-00996-7

**Published:** 2020-09-25

**Authors:** Aduragbemi Banke-Thomas, Mobolanle Balogun, Ololade Wright, Babatunde Ajayi, Ibukun-Oluwa Omolade Abejirinde, Abimbola Olaniran, Rokibat Olabisi Giwa-Ayedun, Bilikisu Odusanya, Bosede Bukola Afolabi

**Affiliations:** 1 LSE Health, London School of Economics and Political Science, WC2A 2AE, London, UK; 2 Centre for Reproductive Health Research and Innovation, Lagos State University College of Medicine, Ikeja, Lagos, Nigeria; 3 Department of Community Health and Primary Care, College of Medicine, University of Lagos, Idi-Araba, Lagos, Nigeria; 4 Department of Community Health and Primary Health Care, Lagos State University College of Medicine, Ikeja, Lagos, Nigeria; 5 Centre for Global Child Health, The Hospital for Sick Children (SickKids), Toronto, Ontario Canada; 6 Dalla Lana School of Public Health, University of Toronto, Toronto, Canada; 7 Department of Population Health, London School of Hygiene and Tropical Medicine, London, UK; 8 Maternal and Child Centre, Ifako Ijaiye General Hospital, Ifako-Ijaiye, Lagos, Nigeria; 9 Department of Obstetrics and Gynaecology, Olabisi Onabanjo University Teaching Hospital, Sagamu, Ogun Nigeria; 10 Department of Obstetrics and Gynaecology, College of Medicine of the University of Lagos, Idi-Araba, Lagos, Nigeria

**Keywords:** Nigeria, Africa, Megacity, Emergency obstetric care, Travel, Urbanisation

## Abstract

**Background:**

The consequences of delays in travel of pregnant women to reach facilities in emergency situations are well documented in literature. However, their decision-making and actual experiences of travel to health facilities when requiring emergency obstetric care (EmOC) remains a ‘black box’ of many unknowns to the health system, more so in megacities of low- and middle-income countries which are fraught with wide inequalities.

**Methods:**

This in-depth study on travel of pregnant women in Africa’s largest megacity, Lagos, is based on interviews conducted between September 2019 and January 2020 with 47 women and 11 of their relatives who presented at comprehensive EmOC facilities in situations of emergency, requiring some EmOC services. Following familiarisation, coding, and searching for patterns, the data was analysed for emerging themes.

**Results:**

Despite recognising danger signs, pregnant women are often faced with conundrums on “when”, “where” and “how” to reach EmOC facilities. While the decision-making process is a shared activity amongst all women, the available choices vary depending on socio-economic status. Women preferred to travel to facilities deemed to have “nicer” health workers, even if these were farther from home. Reported travel time was between 5 and 240 min in daytime and 5–40 min at night. Many women reported facing remarkably similar travel experiences, with varied challenges faced in the daytime (traffic congestion) compared to night-time (security concerns and scarcity of public transportation). This was irrespective of their age, socio-economic background, or obstetric history. However, the extent to which this experience impacted on their ability to reach facilities depended on their agency and support systems. Travel experience was better if they had a personal vehicle for travel at night, support of relatives or direct/indirect connections with senior health workers at comprehensive EmOC facilities. Referral barriers between facilities further prolonged delays and increased cost of travel for many women.

**Conclusion:**

If the goal, to leave no one behind, remains a priority, in addition to other health systems strengthening interventions, referral systems need to be improved. Advocacy on policies to encourage women to utilise nearby functional facilities when in situations of emergency and private sector partnerships should be explored.

## Plain English summary

Many pregnant women in sub-Saharan Africa experience huge challenges in accessing critical maternal health services when in situations of emergency. The challenges of accessing these critical services, otherwise known as emergency obstetric care, is even greater in highly populated cities like Lagos. In this study, we interviewed 47 pregnant women who presented at public hospitals in emergency situations, and 11 of their relatives, to understand their decision-making processes and experiences in such situations.

We found that pregnant women mostly recognise pregnancy danger signs. However, they are often faced with conundrums on “when”, “where” and “how” to reach the hospital in an emergency. While the decision-making process is a shared activity amongst all women, the available options vary depending on their socio-economic status. Women preferred to travel to facilities deemed to have “nicer” health workers, even if these were farther from home. Reported travel time varied in daytime and at night. Many women reported facing remarkably similar travel experiences, with varied challenges faced in the daytime (traffic congestion) compared to night-time (security concerns and scarcity of public transportation). However, the extent to which this experience impacted on their ability to reach facilities depended on their capacity to make their own choices and support systems around them. Referral barriers between facilities further prolonged delays and increased cost of travel for many women.

We concluded that reaching hospitals in emergency situations should not be left to chance. Deliberate efforts to empower women and strengthen hospital referrals will make a difference.

## Background

**Fig. 1:**
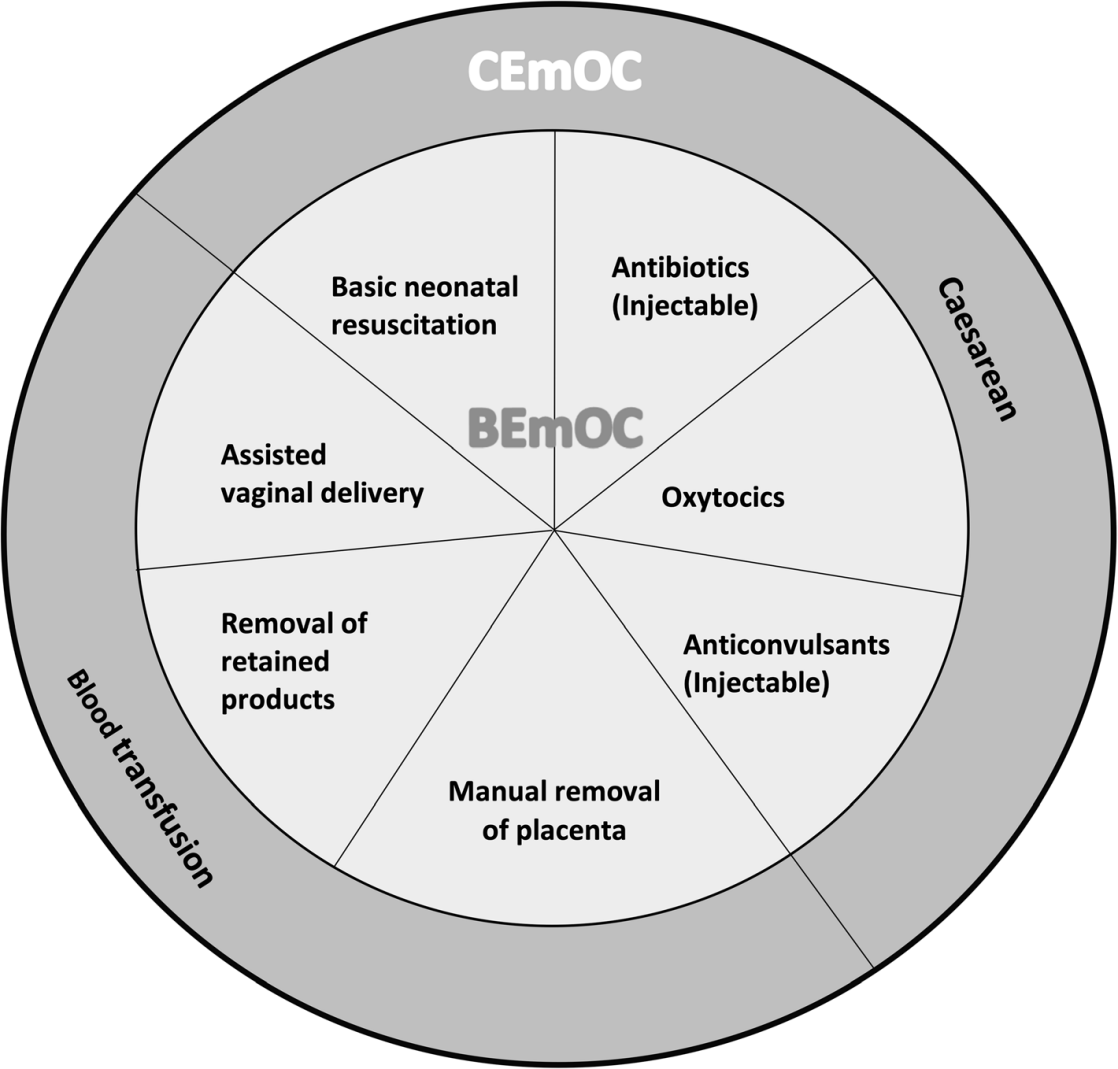
Components of Emergency Obstetric Care. BEmOC: Basic Emergency Obstetric Care; CEmOC: Comprehensive Emergency Obstetric Care

Despite a 29% reduction in global maternal deaths from 1990 to 2015, maternal mortality remains a global health challenge, with about 280,000 women dying every year due to complications of pregnancy and childbirth [1]. Ninety-nine percent of all maternal deaths occur in low- and middle-income countries (LMICs), with sub-Saharan Africa (SSA) accounting for 66% of these deaths (200,000) [1]. Nigeria alone contributes 33% of the maternal deaths (65,000) in SSA [1] despite accounting for only 18% of the region population [2]. Evidence suggests that emergency obstetric care (EmOC) provided by skilled health personnel reduces maternal mortality by as much as 50% and stillbirths by 45–75% [3]. EmOC, as described by the World Health Organization, is a care package made up of nine clinical and surgical interventions. Seven of these nine interventions comprising the administration of parenteral antibiotics, uterotonic drugs, parenteral anticonvulsants, manual removal of placenta, removal of retained products of conception, assisted vaginal delivery and neonatal resuscitation are classified as basic emergency obstetric care (BEmOC). In addition to BEmOC services, blood transfusion and caesarean section make up comprehensive emergency obstetric care (CEmOC) [4] (Fig. 1).

Delays in decision-making to seek care (first delay), travel to reach health facilities (second delay), and provision of appropriate care upon arrival at the facility (third delay) impede EmOC access and have long been associated with increased risk for maternal deaths [5]. This three-delay model, as it is commonly referred to, has been useful in supporting the identification of context-specific challenges that women face in accessing EmOC [6]; however, it has also been critiqued for its simplicity [7]. Across the three delays, LMIC health systems have opportunities to interact directly with women requiring EmOC during the first and third delay phases. For example, skilled health personnel can leverage ante-natal clinic attendance and community outreaches to encourage women to seek facility-based delivery in an emergency [8–10], thereby forestalling the first delay. In addition, they can provide the care that the women require on arrival at health facilities in a timely fashion [3, 11], which reduces the third delay. However, the travel trajectory between home and facility is a ‘black box’ in many LMIC health systems, as women are on their own or with their relatives, and expected to find their way to health facilities in emergency situations [12]. Ultimately, how quickly a pregnant woman in an emergency situation can arrive at a hospital has huge implications for timeliness of service delivery and ultimately on outcomes for her and her unborn child [13].

Pregnant women in emergency situations may experience a complicated travel path to reach health facilities [14]. This journey can be even more complicated in a megacity - defined as a metropolitan area with a population of more than 10 million people [15]. Megacities are fraught with increasing socio-economic vulnerability due to mounting poverty, socio-spatial, political and institutional fragmentation and often extreme forms of segregation, disparities, and violence [16, 17]. But even within megacities, wide inequalities warrant a need for further categorisation into slums and non-slum areas [18]. In addition, varying geographical terrains, including land and water, pose different travel challenges and barriers to access for residents [16]. These considerations point to the non-homogenous nature of megacities and with their rapid emergence in SSA and LMICs more broadly, the need to uncover what goes on during the second delay is more critical than ever.

In SSA, two cities - Kinshasa, Democratic Republic of Congo and Lagos, Nigeria have emerged as megacities with population estimates of 12 and 21 million respectively [15]. However, according to a 2018 systematic review that looked at access and utilisation of EmOC at health facilities in SSA [19], no study has been conducted in Africa’s megacities and those that have been done in urban settings, have either been quantitative studies or qualitative studies with health care providers. Only two studies recruited the women who actually travelled to the health facilities [20, 21], and neither captured experiences of women in reaching facilities nor did they explain their decision-making process in emergency situations. This paper attempts to fill this gap in the literature. Specifically, our objective was to describe in granular details the travel experiences of pregnant women in emergency situations in reaching health facilities within a megacity, using Lagos, Nigeria as a case study. It is expected that insights from this paper will be relevant for planning service delivery and policy initiatives in similar settings.

## Methods

### Setting

Lagos State is the economic nerve centre of Nigeria and arguably the most industrialised part of the country. It is divided into 20 Local Government Areas (LGA). The coastal state has a mix of remote and built-up areas, metropolis and slums, land and riverine areas with a variety of travel options including road, water and rail. The most popular mode of travel is by road.

As per the most recent National Demographic and Health Survey, in Lagos 77% of women deliver in health facilities [22]. The state’s estimated maternal mortality ratio (MMR) is 450 (95% CI 360:530) per 100,000 live births [23], compared to the most recent national estimate of 512 maternal deaths per 100,000 live births [22].

**Fig. 2:**
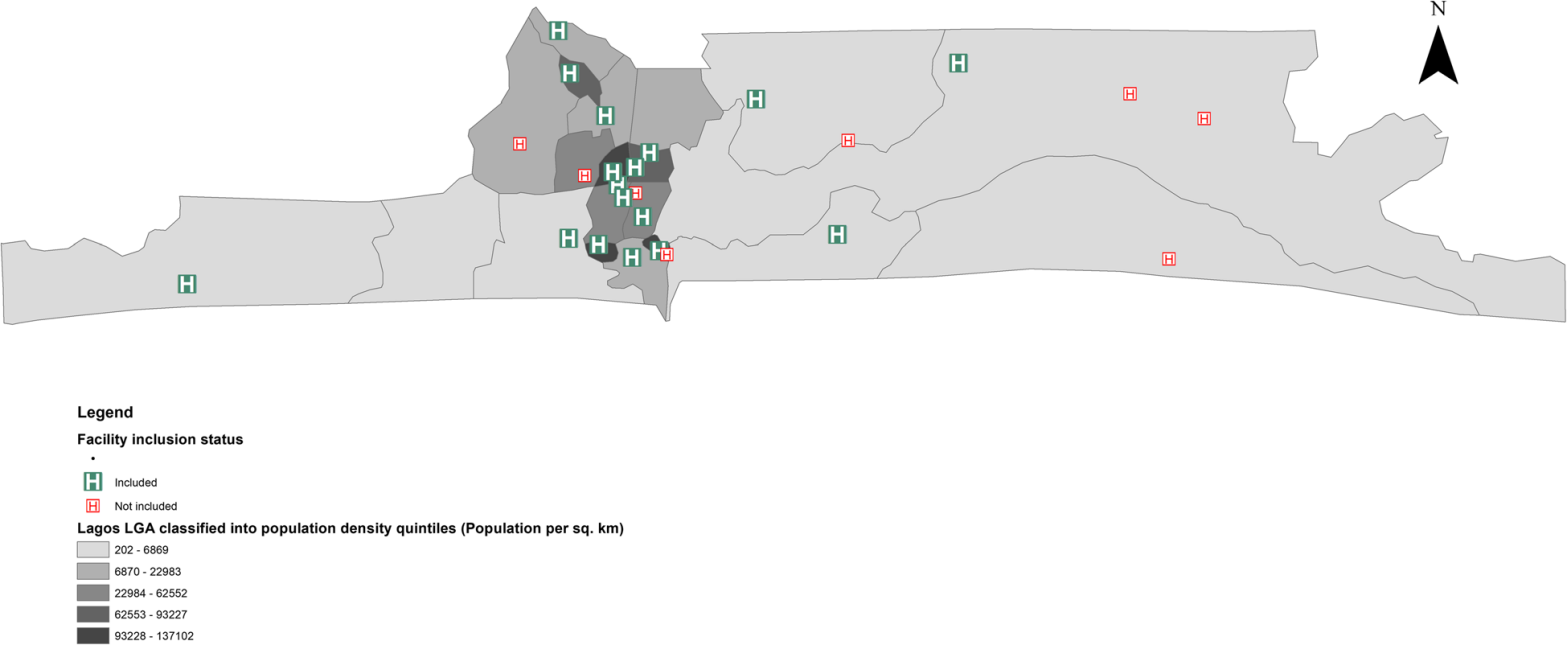
Public sector CEmOC facilities in Lagos highlighting those included in this study

All 24 government-owned (state and federal) health facilities with capacity to provide CEmOC in Lagos were eligible to participate in this study (Fig. 2). We focused only on public sector CEmOC facilities, as they form the bedrock of universal health coverage in Africa [24]. Using criterion sampling [25], 16 of the 24 eligible facilities were purposefully selected for this study. Criteria upon which facilities were sampled included type of urban settlement where the facility is located (town/suburb/city) and type of urban residential area served (slum/non-slum). This was done to ensure maximum variation of travel scenarios of women to facilities.

### Sampling and data collection

Women and their relatives, 18 years or older, were purposively and opportunistically sampled while ensuring heterogeneity of interviewees in order to guarantee variation of characteristics based on age, presenting complaint, parity and socio-economic status (SES). We verified these characteristics by reviewing case notes of the women along with the medical team before approaching them. We borrowed insights from studies conducted in Nigeria to classify women into low, medium or high SES, considering their level of education, employment status and family monthly income [26, 27]. Our sample’s variability allowed for an examination of how these characteristics influence travel in emergency situations. Women were approached in the post-natal or recovery wards following permission of the medical team managing their care.

Five interviewers (AB-T, MB, BA, ROG-A, and BO), trained in qualitative research methods and able to communicate effectively in English and the local languages principally spoken in Lagos (*Yoruba* and *Pidgin English*), conducted the in-depth interviews (IDI). We employed IDIs for data collection as they are ideal for preserving confidentiality, especially for a sensitive and emotive issue as the one explored in our research and also allowed a robust exploration of each woman’s experience [28]. Data was collected between September 2019 and January 2020.

Semi-structured interview guides with open-ended questions developed originally in English, then translated to *Yoruba* and *Pidgin English*, and subsequently back translated, and tested with non-recruited respondents were used. Specifically, the guide explored issues around planning for travel in situations of emergency, decision-making in such situations, experiences of travel on the day of emergency, challenges and how they were dealt with as well as perceived impact of travel on pregnancy outcomes. Efforts were made to ensure that the interviewees felt comfortable enough to express themselves and behave naturally through establishment of rapport upfront. Interviews lasted between 20 and just over 40 min and were conducted in private rooms of the wards, or in secluded bed areas of the women ensuring that conversations were discrete and private. Comments made by interviewees were repeated to them to verify that the interviewer’s understanding conveyed their intended meaning. All interviews were audio-recorded using two voice recording devices to provide back up in case of any malfunction and reflective notes capturing non–verbal events were taken. Interviewer perspectives on the subject matter were set aside before the interviewees and revisited routinely throughout the data collection period during team meetings, in line with guidance from the literature on bracketing [29]. Data collection continued until thematic saturation was achieved.

### Data analysis

Following verbatim transcription of audio-recordings, we followed Braun and Clarke’s six-steps for thematic analysis [30]. To ensure analytical rigour, each transcript was read over by the researcher who conducted the interview and the lead author (AB-T), preliminary thoughts on the transcript were collected during a debrief session after interviews had been completed in each facility. These sessions provided an opportunity to check understanding, reflect on emerging themes, identify novel lines of enquiry, and interrogate any peculiarities in the data.

Initial codes were generated and applied to the transcripts on subsequent readings. An inductive analytical approach was taken in generating the codes facilitated by a Computer Assisted Qualitative Data Analysis Software, NVivo 11 Plus (QSR International, Memphis, USA). Codes that shared similar meanings were grouped together, and these groupings were reviewed and revised until the coded data had been organised into a set of internally consistent themes. Emerging themes were reviewed in tandem with transcripts to check that they accurately captured content, new understanding tested and alternative explanations sought [31]. In describing the emerging themes, constant comparison and deviant case analysis techniques were applied. To achieve this, we leveraged our initial categorisation of included facilities and women to explore similarities and differences between interviewees.

﻿We reported our study following the Standards for Reporting Qualitative Research reporting guidelines [32] (Completed checklist attached as Additional file 1).

### Ethical considerations

Participation was entirely voluntary. Women and their relatives could withdraw from the study at any time. Informed consent was obtained verbally from those who agreed to take part, and no financial incentive was offered. Those who agreed to partake but were deemed emotionally fragile post-delivery were excluded and offered professional psychological support and counselling. In addition, anonymity of patients and facilities from which they were recruited was maintained in reporting this study. Ethical approval for the study was obtained from the Research and Ethics Committee of the Lagos State University Teaching Hospital and Health Research and Ethics Committee of the Lagos University Teaching Hospital.

## Results

In all, 47 women and 11 of their relatives who presented at CEmOC facilities in situations of emergency, requiring some EmOC services were recruited for this study. Age of the women ranged from 21 and 40 years old. Table 1 shows the sociodemographic and obstetric profile of the interviewees.

**Table 1:** Sociodemographic and obstetric profile of interviewees

Facility code	Age	Marital status	SES classification	Parity	Presenting complaint(s)	Relative interviewed
B	24	Married	Middle	Primipara	High blood pressure and preterm delivery	No
	39	Married	Low	Multipara	Obstructed labour	No
	37	Married	Low	Multipara	Footling breech	No
D	32	Married	Low	Multipara	Bleeding (Primary Post-partum haemorrhage)	No
	29	Single	Middle	Primipara	Bleeding (Ectopic pregnancy)	No
E	28	Married	Low	Multipara	High blood pressure and not feeling foetal movement (Intra-Uterine Foetal Death)	No
	24	Single	Middle	Primipara	Incomplete abortion	No
F	23	Married	Low	Primipara	High blood pressure, presented unconscious	Husband
	21	Single	Low	Primipara	Bleeding secondary to induced abortion	Mother
	32	Married	Low	Multipara	Fever and not feeling foetal movement	No
H	31	Married	Middle	Primipara	Obstructed labour	Husband
	33	Married	High	Multipara	Labour not progressing (Obstructed labour)	Sister
	29	Married	Middle	Primipara	High blood pressure and obstructed labour	No
	29	Married	Middle	Multipara	Bleeding (Ectopic pregnancy)	No
I	28	Married	High	Primipara	High blood pressure and severe headache	No
	27	Married	High	Multipara	Not feeling foetal movement	Husband
	32	Married	Middle	Multipara	Dizziness and not feeling foetal movement (Pre-eclampsia, Intra-Uterine Foetal Death)	No
J	33	Married	High	Multipara	Feeling faint and sharp epigastric pain, High blood pressure (Pre-eclampsia)	No
	34	Married	Middle	Multipara	High blood pressure	No
M	25	Married	Middle	Primipara	Prolonged labour	No
	30	Married	Low	Multipara	Profuse bleeding following spontaneous abortion	Sister
	31	Married	Middle	Multipara	Inevitable abortion	Mother-in-law
O	26	Married	Middle	Primipara	High blood pressure and severe headache	No
	29	Single	Low	Multipara	Obstructed labour	No
	22	Married	Low	Primipara	High blood pressure and severe headache	No
	21	Single	Low	Primipara	Bleeding (Ante-Partum Haemorrhage)	Mother
R	33	Married	High	Multipara	Obstructed labour/ Complications post caesarean section	No
	23	Single	Low	Primipara	Fever (Sepsis) and not feeling foetal movement (Intra-Uterine Foetal Death)	No
	40	Married	Low	Grand-Multipara	Preterm labour and multiple gestation	No
	27	Married	Medium	Primipara	Newborn requiring incubator	No
S	35	Married	Middle	Multipara	Obstructed labour	No
	35	Married	High	Primipara	High blood pressure (Pre-eclampsia)	No
	28	Married	Middle	Primipara	Prolonged labour	Sister
	19	Single	Low	Primipara	Bleeding (Induced abortion)	No
T	39	Married	Middle	Grand-Multipara	High blood pressure	No
	25	Married	Middle	Primipara	Abdominal pains	No
	33	Married	Low	Multipara	Bleeding (Abdominal pregnancy)	No
U	25	Married	Low	Primipara	Bleeding and abdominal pains (Ectopic pregnancy)	No
	27	Married	Low	Multipara	High blood pressure (Pre-eclampsia)	No
W	30	Married	Middle	Primipara	High blood pressure (Pre-eclampsia)	No
	32	Married	Middle	Multipara	Inevitable abortion	No
X	28	Married	Middle	Multipara	Prolonged labour	Mother-in-law
	32	Married	Middle	Multipara	Headache, vomiting, high blood pressure	Husband
	39	Married	Low	Primipara	Prolonged labour	No
	31	Married	Middle	Multipara	Foetal distress and Breech delivery	No
Y	29	Separated	Middle	Primipara	Headache, epigastric pain, and swelling	No
	28	Married	High	Primipara	Bleeding (Ante-partum haemorrhage)	No

The key themes with subthemes that emerged are described under three headings below (Table 2).

**Table 2:** Themes and sub-themes

Themes	Sub-themes
Decision-making in emergency situations	• Decision of when to leave for the hospital depends on perceived urgency and risk of travel
	• Decision of where to go depends on facility proximity, perceived responsiveness and connections
	• Decision on how to travel was about availability
Travel experiences of pregnant women in emergency situations	• Traffic conditions, bad roads, security concerns and travel cost are real challenges for all
	• Socio-economic status and support of relatives help to optimise travel experience
Reaching another facility after being referred	• Referrals increase travel time and delays in service delivery
	• Ambulances are not the magic bullet for referral

### Decision-making in emergency situations

#### Decision of when to leave for the hospital depends on perceived urgency and risk of travel

Women in our study did not typically make travel plans for emergencies that could occur in pregnancy before they found themselves in situations of emergency, irrespective of their ante-natal clinic attendance. Most of the reported lack of preparation for an emergency was founded on religious beliefs. One 30-year-old, middle SES, primiparous woman, who presented with increased blood pressure at Facility W (suburb, slum) said, “*Planning [for emergency] means I am expecting a problem. I know that women can have emergencies in pregnancy, but I know my own cannot be like that. God knows why this one happened*”. However, irrespective of age, SES, or parity, women generally acknowledged that they recognised symptoms such as sharp abdominal pain, bleeding, severe headache, fever, not feeling foetal movements, or appearance of foetal parts from the introitus as symptoms that meant they needed to get help. While those who had attended ante-natal clinic in some health facility (public or private) appeared to have a better sense of urgency, those who had not registered in a hospital before they found themselves in emergency situations (i.e. un-booked women) appeared slower in making the decision to travel to a facility. One relative of an un-booked woman said:

*“On her arrival, first day, second day, she told me to give her a cloth. I asked why and she said she was seeing something that looked like her menses coming from her vagina … On the third day, we were waiting by the roadside... she just suddenly saw blood and then she alerted me. So, I said she should go inside the house into the bathroom while I go get some water for her. Before I got back to her, the paint bucket that she sat on was filled with blood. Then she said she was feeling dizzy. So, I told her to allow me quickly purchase milk for her. Before I got back, she had already fainted. I thank God, after we poured water on her, screamed her name and also by the help of God, she regained consciousness (Sighs!) Then I started shouting, “Where is a hospital? Where do I take her to”? … ”*. Relative (Sister) of 30-year-old woman, low SES, multipara, spontaneous abortion that had been managed by a traditional birth attendant out-of-state and now presented in the hospital with profuse bleeding [Facility M (suburb, slum)].

However, if the emergency occurred late at night, or there were concerns about security and safety on the roads, some women who recognised that they were in an emergency situation needing facility-based care waited till daybreak to commence their journeys. Women who reported waiting till morning were typically those in early pregnancy (first trimester). However, despite safety concerns, women in later stages of pregnancy (second and third trimesters) reported being more focused on the need to be delivered of the baby than being concerned about security.

“*I was at home and I was having severe pains from about 2 a.m. that morning. I woke my husband up immediately, but because of our concerns with safety at that time, we decided to leave for the hospital later at about 6 a.m. … ”* 29-year-old, medium SES, multipara, with bleeding and abdominal pain [Facility H (suburb, non-slum)]

“*My husband was saying it is too late and it would not be safe for us to be on the road. But as a pregnant woman, when you are in labour, you are not thinking about anything. You just want them to remove the thing [the baby]*”. 33-year-old, high SES, multipara, labour not progressing [Facility H (suburb, non-slum)]

The choice on when to commence travel did not appear to be influenced by which facility they intended to visit or where it was located. However, there were a few women who knew that they were in an emergency situation but decided to make stopovers along the way to pick up “experienced”, “needed” or “helpful” relatives like mothers and mothers-in-law or go for shopping for items needed for their hospital admission. The choice to do this did not appear to be dependent on SES or parity. A 28-year-old, medium SES, multipara, who presented with prolonged labour said, “*we had to go pick my mother-in-law at [her place] before we started coming to this place [hospital]*”. Similarly, another 33-year-old, low SES, multipara, presenting with heavy bleeding [Facility T (suburb, non-slum)] said, “*Before I came, I went to market to buy a few things that I thought I needed [in the hospital], before coming here [the hospital]. When I got here, they said they can never let me go, that my BP [blood pressure] was so high at 250 plus [systolic pressure]*”.

#### Decision of where to go depended on facility proximity, perceived responsiveness and connections

For the decision on where to go in situations of emergency, women reported that several factors were considered. For some women, the decision on where to go in situations of emergency was made a priori based on the CEmOC facility where they received antenatal care (ANC). For such women, their choice of facility was principally based on proximity as well as community or a relative’s perception of the facility. One 29-year-old, medium SES, primipara, who presented with bleeding as a result of an ectopic pregnancy [Facility Y (suburb, slum)] noted that, “*My sister had a baby here and she recommended it … So, I did not mind registering here. Secondly, this place is near to my house, so just in case something happened, I can quickly get here. And you see, I needed it*”. However, for many women in our sample, perception trumped proximity. For such women, the negative perceptions that they had of specific facilities prevented them from attempting to travel to nearer facilities in situations of emergency. A 24-year-old, middle SES, primiparous who presented with high blood pressure in pregnancy [Facility B (suburb, slum)] said, *“[Facility D (suburb, slum)] was closer to me, but I heard that they exchange people’s babies when they are born … So, me I said, I am not going there*”. A positive perception made women in situations of emergency travel further because another facility was deemed to “*be better*”, “*provide good care*”, or as having “*caring staff*”. The case for going to a farther hospital was reinforced when influential relatives (husbands and mothers-in-law) or associates (leaders in religious organisations) recommend those facilities. One 25-year-old, low SES, primiparous, bleeding and abdominal pains [Facility U (suburb, slum)] said, “*It was my mother-in-law that suggested that we use this hospital. She told me that is the hospital she uses, and other relatives also deliver here. And that I will receive good care*”. When probed further, we found that there were three closer facilities that were alternatives to her. Another woman said:

“*It was my husband and brother that brought me here … we passed some [three] hospitals before we got here, but everyone knows that the people here are very caring*”. 33-year-old, high SES, multipara, feeling faint and sharp upper abdominal pain [Facility J (suburb, non-slum)]

Health insurance coverage in specific facilities was an additional reason given by women in deciding where to go. One 32-year-old, medium SES, multiparous, who presented with dizziness and was managed for pre-eclampsia, lost her baby while going to a more distant facility where her health insurance would be accepted. In addition, women talked about having connections (i.e. privileged access) to senior health workers in the facility as a deciding factor. In fact, pregnant women in situations of emergency reported altering their initial choice of where to go while in transit based on advice received from influential people. The underlying motivation appeared to be the reassurance that they would receive the emergency care that they needed urgently upon their arrival at the facility. Use of connections was particularly reported amongst high and medium SES women. In a respondent’s words:

“*My husband and I agreed that I should do my antenatal at [Facility E* (suburb, non-slum)*]. But when my husband called his boss that night, he advised that we are better going to [Facility I]. He knew the ògá [i.e. medical director] there, and he will call them, so that we get the necessary care*.” 27-year-old, high SES, multipara, not feeling baby movement [Facility I (suburb, slum)]

#### Decision on how to travel was about availability

Women in our study did not report their ‘how to travel’ i.e. mode of transportation as a matter of choice; it was a given based on availability. For those who owned cars, this was used in all instances. Some who did not have their own cars relied on partners to first make a trip to loan a car from relatives or friends or to hail a taxi through mobile apps, direct calls to known taxi drivers or through roadside taxis. However, for those who could not access any of the aforementioned means, the next two popular options were public “kękę” (commercial tricycle) and “okada” (commercial motorcycle), with general preference for the former. A 26-year-old, low SES, primipara, who presented with high blood pressure and severe headache [Facility O] said, *“I prefer kękę, it is safer than okada. You know it is emergency. So, with kękę, at least there is cover, because someone can still fall from okada, while dizzy”.*

However, for some who live in riverine areas, the sole mode of transportation was by boat - either one that they owned or one that was used by the public. Nevertheless, this mode of transport still needed to be combined with land travel to reach facilities in situations of emergency.

“*The only way to come here was to first take boat to the other side and then take kękę from there. We don’t have hospital on our side. My husband has his own boat that he uses for fishing. So, with the help of his brother, they brought me to this hospital …* ”. 32-year-old, low SES, multipara, with fever and not feeling foetal movement [Facility F (town, non-slum)].

### Travel experiences of pregnant women in emergency situations

#### Traffic conditions, bad roads, security concerns and travel cost are real challenges for all

The deplorable conditions of the roads only further prolonged travel. One 23-year-old, low SES, primiparous woman who presented at a facility located in a non-slum town with raised blood pressure [Facility F (town, non-slum)] said that, “*Normally that journey will take maybe 20 minutes, because it is a highway, but my husband said it took us over 45 minutes … It is under construction at the moment*”.

Another woman who travelled to reach a facility in a suburb, slum area said:

“*I took taxi... I was worried when I was being transported on the roads. There were so many potholes. I was just holding myself. Even though it was just a short journey, maybe like six minutes, from my house to the hospital. But I felt like I was going to die.*” 39-year-old, low SES, multiparous, obstructed labour [Facility B (suburb, slum)]

Women who travelled at night in towns and suburbs reported difficulty in finding transportation options, being stopped by police en route and the facility gate being closed upon arrival and only opened after beckoning to the security persons at the gate.

“ *… This was now after 11 p.m. When the doctor came, he said they could not handle it and then referred me to this hospital. We looked for kękę, we couldn’t find. When we finally found one, we got on the road but there were so many policemen on the road stopping us. Though they let us go when they saw me. We got here at about 1 a.m. the next morning. The gate was locked, but when my husband knocked, they let us enter. Thank God for my life. I could have died. It is the water that poured, the container did not break*”. 37-year-old, low SES, multipara, with a footling breech [Facility B (suburb, slum)]

Travel with personal cars was described by women as being “at no additional cost, except for the petrol purchased”. However, for low SES women, many complained about the expensive cost of travel, for which they had to sometimes “borrow money”, “plead with the driver”, “take okada” (which was deemed cheaper), or “walk some distance to save money”. It was not unusual for women to use multiple transport means to reach facilities. A relative (mother) of a 21-year-old, low SES, primiparous, who presented with bleeding as a result of antepartum haemorrhage [Facility O (town, non-slum)] submitted that, *“We spent ₦200* (US$0.55) *from the house to the bus stop in kękę. Then from the bus stop to the main stop in the area of the hospital on a 30-minute bus ride, ₦200* (US$0.55) *and then okada to the hospital, ₦100* ($0.27) *per person”*. Thirty-three of the 47 women in our study who took public transport reported spending between ₦300 ($0.82) and ₦7,500 ($20.60) to reach facilities in Lagos. When disaggregated, women who travelled within towns or suburbs to reach CEmOC facilities in cities using public transport appeared to spend more compared to those who travelled within the cities. Twenty-seven of the women who reside in suburbs spent between ₦1,200 ($3.30) and ₦7,500 ($20.60) while six of their counterparts who reside in urban areas spent between ₦300 ($0.82) and N2,500 ($6.87). Irrespective of settlement type, women reported that drivers hiked fares if they were carrying luggage with them or if they were travelling at night.

“*We took taxi to [this hospital] when I started having problems … It would have been ₦2,000* ($5.49) *in the morning but at that time of the night they said ₦5,000* ($13.74)*. He even said he could have charged us more but because he is of the same ethnicity as my husband, he allowed us pay less*”. 32-year-old, low SES, multiparous, with bleeding [Facility D (suburb, non-slum)]

#### Socio-economic status and support of relatives help to optimise travel experience

Women and their relatives reported varying experiences when they set out to CEmOC facilities. Experiences varied with the time of travel, as well as the terrain navigated to reach the facility. Irrespective of whether the facility was located in a town, suburb or city, women who lived within one or two streets away from CEmOC facilities had about 5–10 min of travel using taxis or their personal cars. Across the entire sample of 47 women, estimated time of travel was between 5 min to almost 4 h in daytime and between five and 40 min at night. At night, there did not appear to be much variation in travel time to reach facilities based in towns, suburbs or cities, irrespective of how close or far women lived from the facilities. Neither was there much variation in reported travel time to reach facilities serving mostly non-slum (5–30 min) and slum (15–40 min) populations. Women reported that time of travel in the afternoon could be between two and six times more if there was heavy traffic. In such conditions, women reported resorting to the use of extreme measures, including driving on illegal lanes, facing oncoming vehicles or abandoning the cars they were in to flag down a motorcycle. One particular woman’s experience trying to reach a facility located in a suburb, non-slum area captures some of these extremes:

“*The experience of getting to this place was terrible. I first went to a private hospital. From my house to that hospital, typically one hour no traffic. But we needed to be in hurry, we had to take the BRT [Bus Rapid Transit] lane. A policeman stopped us but the church member who was carrying me explained to him that he was carrying a pregnant woman, he then let us pass. But the traffic was still too much. We had to turn back on the road to drive ‘one-way’ to reach this hospital.*” 35-year-old, medium SES, multipara, with obstructed labour [Facility S (city, non-slum)].

In other instances, women reported using motorcycles which allowed them “beat the traffic”. However, they highlighted the discomfort and additional pain they had to endure with this mode of travel, compounded by the deplorable conditions of many of the roads. This road condition concern was shared by women irrespective of the time of the day that they travelled, the length of the trip or the location of the facility. One woman who presented at a facility located in a suburb and non-slum area said:

“*I had to take okada from my house to the primary health centre, because I knew this would be faster, especially as the road is not tarred and there is usually plenty traffic on it. In all, the journey was about 30 minutes.... On my way going, when the bike enters a pothole, I will shout because I was in pain. But I didn’t have a choice!*” 31-year-old, medium SES, primipara, with prolonged labour [Facility H (suburb, non-slum)]

Women highlighted that having relatives with them along the way to reach CEmOC facilities was “very helpful”, “important”, and “most needed”. Relatives reported that they had to try all they could to support and manage their loved ones in situations of emergency and in some cases, they had to treat them any way that they knew. A relative (husband) of a 23-year-old, low SES, primiparous, who presented unconscious at Facility F (town, non-slum) said, *“She definitely does not know how she got here. She was gone [unconscious]! I had to put a spoon in her mouth [a local technique done to unconscious patients based on the belief that clenching of teeth signifies end of life]”.*

### Reaching another facility after being referred

#### Referrals increase travel time and delays in service delivery

Seventeen women required referral to a CEmOC facility, as the BEmOC or CEmOC facility they initially presented to could not provide the necessary care. Service delivery limitations included lack of bed space or incubators for their newborns. Some of these women had to move through multiple facilities in order to access the required care. A 24-year-old, medium SES, primipara, who presented with high blood pressure and pre-term delivery in Facility B (suburb, slum) stated “*I registered with one matron in my community and I was placed on blood pressure medications. But the baby was still small and needed incubator. So, she referred me to [Facility D (suburb, non-slum)] … But when I got to that facility, the doctor said, they don’t have any space for incubator, so they now referred me here [Facility B]”.*

#### Ambulances are not the magic bullet for referral

Ambulances, which are expected to be available in all state-owned CEmOC facilities, are typically used to transport patients from facility to facility in situations of emergency. When available and not in use and if there was no traffic, women referred in hospital ambulances found it to be advantageous, irrespective of urban settlement type. The reason given by women for such perception was that an ambulance provides supportive equipment and hospital drivers are more conversant with the directions to the referral hospital. However, some women reported that even though they had to pay to be transported in the hospital ambulance, it did not help to reach the referral facility any quicker. The women attributed this to the lack of regard for ambulances by other road users; there does not appear to be a culture of motorists giving way to ambulances on Lagos roads.

“*The road was free … maybe 15 minutes max to reach here. It (the ambulance) had all I needed – oxygen, life support, everything! And then I have never been here in my life, so not like I even know my way to this place. It was a smooth process!*” 33-year-old, high SES, multiparous, with complications post-caesarean [Facility R (city, non-slum)]

“*They used their ambulance, which they told me to pay for. I paid ₦3,000 ($8.25). I left the hospital at 4 p.m. and I got here around 9 p.m. The traffic was really bad that day!! The ambulance did not help us to get here faster. Even people in their private car were dragging the road with us. They don’t even want to know if somebody is dying or something … If it was in my state, people will give way to ambulance*”. 24-year-old, single, primipara, with incomplete abortion [Facility E (suburb, non-slum)]

Some women were also referred to state-owned CEmOC facilities from primary health care facilities or private hospitals. However, ambulance use for referral in these facilities was variable. In many instances, women were referred and expected to figure out on their own how to reach the referral facility. One 27-year-old, medium SES, primiparous, who was referred from a health centre with her newborn requiring incubator [Facility R (city, non-slum)] said, *“They did not even mention ambulance. So, my husband had to get taxi. We travelled for almost three hours from the health centre to get here”.*

## Discussion

This study set out to critically explore the factors influencing and experiences of travel of pregnant women in situations where they are attempting to access EmOC in Africa’s largest megacity, Lagos. Findings showed that after pregnant women have identified an emergency situation, they are still faced with real conundrums on “when”, “where” and “how” to travel to reach appropriate facilities. While this decision-making is a shared activity amongst all women, the options and the process vary. Our interviews revealed remarkably similar experiences of travel, irrespective of women’s age, socio-economic background, or obstetric history. Nevertheless, the extent to which travel experiences impacted their ability to reach EmOC facilities depended on individual agency and support systems. Due to service delivery limitations, some women had to be further referred, which further prolonged travel and increased cost.

Women in our study recognised danger signs, regardless of their age, SES, obstetric history and presenting complaint. Our findings also showed that being aware of danger signs does not necessarily translate to responding to the urgency of the situation. It is difficult to conclude that decision on when to travel was made more quickly amongst those of higher SES compared to low SES in our study. Though a similar qualitative study conducted in Ghana found that women who lack formal education (a component of SES) tend to delay in recognising danger signs [33]. Our finding points to the reluctance of women facing obstetric emergencies, many of whom are of low SES, in presenting at facilities, when they had not registered for ANC. This might be due to concerns at being chastised for not being booked or the cost associated with facility-based care [34]. On the other hand, some women of high SES also delayed travel either because they felt it was best to travel when it was safer (i.e. during the day) or because they downplayed indications of danger. Women who lost their lives due to complications of pregnancy and childbirth in Gambia have been shown to underestimate the severity of their complication and as such delay travel and care seeking [35]. Our findings indicate that the perception of urgency may be influenced by some symptoms which women perceive as being more severe.

The choice of ‘where’ to go was influenced by proximity for some women, especially when they resided close to facilities. However, for many women, proximity was trumped by family or community perception of the facility, health insurance coverage, connections with highly placed individuals within the health system and the advice of influential people. While a previous study showed that there is a general “positive perception” by women of public CEmOC facilities in Lagos, especially as it relates to the availability of technically sound skilled health personnel [34], concerns remain regarding responsiveness of staff and cost of services. Evidence points to the consensus that women place importance on attitudes of health care providers [36]. While a 2018 systematic review on barriers to EmOC access in sub-Saharan Africa does not point to lack of connections as a barrier [19], women, especially those with high SES, clearly felt that they needed the “added advantage” it provides, as a way of guaranteeing the quality of care.

We found that decision-making on “how” to travel was based on availability. For women of high and medium SES who owned a car, had a relative nearby who owned a car, or had access to and could afford taxis, vehicles were the preferred mode of transport in an emergency. In other LMIC settings, lack of vehicles has been reported as a barrier to accessing EmOC [20, 21, 37–39]. However, the striking finding in our study was the level of risk that women were willing to take when they did not have access to a vehicle. The motorcycle was a common mode of travel for women, especially in remote areas as well as areas prone to significant traffic. However, it is important to bear in mind that beyond the risk posed by these two-wheelers to pregnant women, at least 30% of road traffic accidents have been attributed to them [40]. It was, therefore, good to find that women in our study perceived the tricycle as a safer alternative. This probably complements ongoing efforts to scale up tricycles for supporting pregnant women in emergency in some other parts of the country [41].

Irrespective of the choices that women made, their socio-economic status or obstetric history, many women in our study reported facing significant challenges in travelling to health facilities, with different challenges faced during the daytime (traffic congestion) and night-time (security concerns and scarcity of public transportation). The deplorable conditions of many of the roads was a huge challenge, regardless of the time of day. A 2015 review reported travel time between 10 min and 1 day [42]. Women in our study reported they spent between 5 min and 4 h. This is probably because of the urban nature of our study setting. Women also reported that the traffic combined with bad road conditions could increase travel time as much as 200–600%. These estimates are not too far from those reported in the grey literature, where as much as 800% increase in travel time due to traffic has been reported [43]. For the urban poor in particular, such increases in travel time have been found to be a strong deterrent to seeking EmOC. A study in Bangladesh estimated that for every five-minute increase in travel time to the nearest EmOC facility there is a 30% decrease in the likelihood of delivery at an EmOC facility; favouring home-based care [44]. This ultimately nullifies any drive to scale up facility-based deliveries in the absence of broad systemic and infrastructural changes that address the second delay. In our previously published multi-stakeholder analysis on EmOC access, while the Lagos State government believed that EmOC facilities have been strategically located across the state, several women reported difficulty in accessing facilities [45]. Some have suggested that these constraints could be related to poorly located EmOC services [46] or ﻿insufficient numbers of EmOC facilities within a recommended distance [47].

For night travel, security concerns reported by women is shared by the Lagos population. However, no woman in our study specifically reported this as part of her experience in trying to reach a health facility. The price hike at night is not a unique experience in Lagos. Pregnant women in Ethiopia and Nepal have previously reported feeling financially exploited by transporters [42]. Such exploitation was a concern for women who already found day fares as prohibitive. Several women in sub-Saharan Africa report cost of travel and lack of transport funds as a barrier to EmOC access [19]. However, one key facilitator that improved the experience of travel was having a relative come along on the journey to the facility. Relatives play a significant role in providing emotional, financial and logistic support for which the health system is not structured to provide. A recent qualitative study in Ghana highlighted that sometimes relatives have to play the role of “escort” since health facilities do not have enough staff to escort patients [48].

As previously reported in the literature [35], our study found that even when women made it to a CEmOC facility, referral between facilities further prolonged delays and increased cost of travel. Specifically, some women in our study who arrived at secondary facilities were referred to other facilities, mostly due to lack of bed space or lack of incubators. These referrals appear to be functional and many pregnant women who were referred and transferred by ambulance described the process as efficient. However, referrals from primary and private facilities to secondary or tertiary facilities remain fraught with challenges including lack of ambulances, with women left alone to figure out how they would reach the facilities. Such shortages have been shown to discourage healthcare providers from referring clients [12]. Poor referral systems are huge barriers to accessing EmOC [49]. The Lagos State Ambulance system mostly functions at the secondary to secondary level. However, even when an ambulance service is available, it does not always guarantee quicker transit time. This was mostly attributed to other road users not giving way to ambulances. This issue of driver etiquette has long been raised as one that needs addressing in Lagos in an audit of ambulance effectiveness [50] and was also recently reported to be an issue affecting the EmOC referral system in Ghana [48].

There are clear implications for policy and practice based on our findings, which support the need for governments to target both health systems and the overall SES of women to improve EmOC access. Firstly, the practice of birth preparedness and complications readiness (BPCR) as part of routine ante-natal care in Lagos hospitals [51] needs to be sustained. However, in addition to the danger sign recognition focus of BPCR (“when”), more emphasis needs to be placed on advising pregnant women on “where” and “how” they plan to travel in situations of obstetric emergency [52]. Some women will be yet to commence ANC before they have an emergency, for example, as seen in our study, women with ectopic pregnancies. Sending messages through opinion leaders in the community may be helpful to reach these women. These women can also be reached via mobile phones as was done in Western Kenya [53].

Secondly, there is a need to standardise costs to women for receiving EmOC, expand health insurance coverage and ensure respectful maternity care [54] in public facilities, so that women are not conflicted in their choice-making when in emergency situations. Thirdly, efforts need to be put in place to improve the travel experiences of women while minimising the risk that they may be forced to undertake in situations of emergency. While road improvements will be helpful to state development, it is cost-intensive. Leveraging xisting structures, such as establishing partnerships with specific taxi companies and tricycles, might offer a cost-effective approach. A recent partial ban of tricycles in Lagos [55] may mandate some policy reflections, before this can be considered. Tricycle riders and private taxi drivers can be trained on proper transfer of women in situations of emergency and integrated into the referral process. In addition, indemnity cover to ensure that liabilities that they may incur while transporting women in situations of emergency such as physical damages to their vehicle [12], should be covered. Legal permission to women in emergencies to use bus-only lanes when in actual emergency situations can also help reduce travel time in emergencies. Campaign for attitudinal change of drivers, as it relates to giving way for ambulances would also be helpful. However, this needs to be supported by legislation to ban misuse of such rights by ambulance drivers, ensuring that the siren is only used in situations of emergency.

To our knowledge, this is the first qualitative study that rigorously describes issues around travel of pregnant women in situations of emergency in a LMIC megacity. By seeking to maximise the heterogeneity of the sampled facilities and speaking to different women with varying characteristics within each facility, this study reflects multiple experiences of travel and the effect that the various characteristics could have on travel to reach facilities in an emergency. Nevertheless, this study also has limitations. Firstly, we only focused on women who made it to the facility, excluding those who had emergencies but never made it to the facility. Future studies need to engage with women and their relatives within the community, in order to identify such women and capture their experiences. Secondly, there could have been interviewer bias from the multiple interviewers who engaged the women. However, the use of a standard operating protocol to guide conduct and debriefing sessions minimised such occurrences. In addition, reported time estimates were based on recollection of the women or their relatives, many of whom were in duress while traveling to reach facilities and as such the reliability of their estimates is questionable. However, efforts were made during the interviews to verify travel time estimates by rephrasing travel time related questions in many ways and asking both women and relatives where possible.

## Conclusions

Our study revealed that the delay in travel to health facilities is a real experience of pregnant women in situations of emergency even in a megacity like Lagos. For pregnant women, reaching a facility in emergency situations is usually a matter of life and death. However, the ability to reach such facilities should not be based on chance, connections, or capacity. Every woman in such situation needs to have a fair opportunity to reach a facility and receive the necessary care at such critical moments. If the goal remains to leave no one behind, then in addition to other health system strengthening interventions, referral systems need to be improved, advocacy to encourage women to use their nearest facilities when in situations of emergency and partnerships with private sector need to be explored.

## Supplementary information

**Additional file 1.** Completed checklist.

## Data Availability

All data supporting these findings is contained in this manuscript. There are no restrictions to anonymised data sources. All data collection tools are available upon request.

## Abbreviations

ANC: Ante-Natal Care;
BEmOC: Basic Emergency Obstetric Care;
BPCR: Birth Preparedness and Complications Readiness;
BRT: Bus Rapid Transport;
CEmOC: Comprehensive Emergency Obstetric Care;
EmOC: Emergency Obstetric Care;
IDI: In-depth Interviews;
LGA: Local Government Area;
LMICs: Low- and middle-income countries;
SES: Socio-Economic Status;
SSA: Sub-Saharan Africa

## References

[1] WHO, UNICEF, UNFPA, World Bank Group, UNDP. Trends in maternal mortality 2000 to 2017: estimates by WHO, UNICEF, UNFPA, World Bank Group and the United Nations Population Division. Geneva: World Health Organization; 2019. p. 1–119. Available from: https://www.unfpa.org/sites/default/files/pub-pdf/Maternal_mortality_report.pdf.

[2] The World Bank. Population, total - Sub-Saharan Africa. Data. 2020 [cited 2020 Aug 14]. Available from: https://data.worldbank.org/indicator/SP.POP.TOTL?locations=ZG.

[3] Paxton A, Maine D, Freedman L (2005). The evidence for emergency obstetric care. Int J Gynaecol Obstet.

[4] World Health Organization, United Nations Population Fund, United Nations Children’s Fund, Averting Maternal Deaths and Disabilities (2009). Monitoring emergency obstetric care: a handbook.

[5] Thaddeus S, Maine D (1994). Too far to walk: maternal mortality in context. Soc Sci Med.

[6] Calvello EJ, Skog AP, Tenner AG, Wallis LA (2015). Applying the lessons of maternal mortality reduction to global emergency health. Bull World Health Organ.

[7] Bohren MA, Hunter EC, Munthe-Kaas HM, Souza JP, Vogel JP, Gülmezoglu AM (2014). Facilitators and barriers to facility-based delivery in low- and middle-income countries: a qualitative evidence synthesis. Reprod Health.

[8] Fekadu GA, Kassa GM, Berhe AK, Muche AA, Katiso NA (2018). The effect of antenatal care on use of institutional delivery service and postnatal care in Ethiopia: a systematic review and meta-analysis. BMC Health Serv Res.

[9] Devasenapathy N, Neogi SB, Soundararajan S, Ahmad D, Hazra A, Ahmad J (2017). Association of antenatal care and place of delivery with newborn care practices: evidence from a cross-sectional survey in rural Uttar Pradesh, India. J Health Popul Nutr.

[10] Baatiema L, Ameyaw EK, Moomin A, Zankawah MM, Koramah D (2019). Does antenatal care translate into skilled birth attendance? Analysis of 2014 Ghana demographic and health survey. Adv Public Heal.

[11] Bailey P, Paxton A, Lobis S, Fry D (2006). The availability of life-saving obstetric services in developing countries: an in-depth look at the signal functions for emergency obstetric care. Int J Gynecol Obstet.

[12] Afari H, Hirschhorn LR, Michaelis A, Barker P, Sodzi-Tettey S (2014). Quality improvement in emergency obstetric referrals: qualitative study of provider perspectives in Assin North District, Ghana. BMJ Open.

[13] Ravelli A, Jager K, de Groot M, Erwich J, Rijninks-van Driel G, Tromp M (2011). Travel time from home to hospital and adverse perinatal outcomes in women at term in the Netherlands. BJOG..

[14] Banke-Thomas A, Wright K, Collins L (2019). Assessing geographical distribution and accessibility of emergency obstetric care in sub-Saharan Africa: a systematic review. J Glob Health.

[15] United Nations (2019). World Urbanization Prospects: The 2018 Revision.

[16] Kraas F (2007). Megacities and global change: key priorities. Geogr J.

[17] Jowell A, Zhou B, Barry M (2017). The impact of megacities on health: preparing for a resilient future. Lancet Planet Heal.

[18] Gaur K, Keshri K, Joe W (2013). Does living in slums or non-slums influence women’s nutritional status? Evidence from Indian mega-cities. Soc Sci Med.

[19] Geleto A, Chojenta C, Musa A, Loxton D (2018). Barriers to access and utilization of emergency obstetric care at health facilities in sub-Saharan Africa: a systematic review of literature. Syst Rev.

[20] Echoka E, Makokha A, Dubourg D, Kombe Y, Nyandieka L, Byskov J (2014). Barriers to emergency obstetric care services: accounts of survivors of life threatening obstetric complications in Malindi District, Kenya. Pan Afr Med J.

[21] Ganle JK, Parker M, Fitzpatrick R, Otupiri E (2014). A qualitative study of health system barriers to accessibility and utilization of maternal and newborn healthcare services in Ghana after user-fee abolition. BMC Pregnancy Childbirth.

[22] National Population Commission, ICF International. Nigeria Demographic and Health Survey 2018. Abuja, Nigeria and Rockville, Maryland, USA; 2019. Available from: https://dhsprogram.com/pubs/pdf/FR359/FR359.pdf.

[23] Oye-Adeniran BA, Odeyemi KA, Gbadegesin A, Ekanem EE, Osilaja OK, Akin-Adenekan O (2011). The use of the sisterhood method for estimating maternal mortality ratio in Lagos state, Nigeria. J Obstet Gynaecol (Lahore).

[24] Sachs JD (2012). Achieving universal health coverage in low-income settings. Lancet.

[25] Patton M (2015). Qualitative research and evaluation methods.

[26] Oseni TIA, Odewale MA (2017). Socioeconomic status of parents and the occurrence of pelvic inflammatory disease among undergraduates attending Irrua specialist teaching hospital, Irrua, Edo state, Nigeria. Niger Postgrad Med J.

[27] Oyedeji G (1985). Socio-economic and cultural background of hospitalised children in Ileesha. Niger J Paediatr.

[28] McGrath C, Palmgren PJ, Liljedahl M (2019). Twelve tips for conducting qualitative research interviews. Med Teach.

[29] Fischer CT (2009). Bracketing in qualitative research: conceptual and practical matters. Psychother Res.

[30] Braun V, Clarke V (2006). Using thematic analysis in psychology. Qual Res Psychol.

[31] Marshall C, Rossman GB (1999). Designing qualitative research. Third.

[32] O’Brien BC, Harris IB, Beckman TJ, Reed DA, Cook DA (2014). Standards for reporting qualitative research: a synthesis of recommendations. Acad Med.

[33] Oiyemhonlan B, Udofia E, Punguyire D (2013). Identifying obstetrical emergencies at Kintampo municipal hospital: a perspective from pregnant women and nursing midwives. Afr J Reprod Health.

[34] Wright K, Banke-Thomas A, Sonoiki O, Ajayi B, Ilozumba O, Akinola O (2017). Opinion of women on emergency obstetric care provided in public facilities in Lagos, Nigeria: a qualitative study. Health Care Women Int.

[35] Cham M, Sundby J, Vangen S (2005). Maternal mortality in the rural Gambia, a qualitative study on access to emergency obstetric care. Reprod Health.

[36] Bohren MA, Vogel JP, Hunter EC, Lutsiv O, Makh SK, Souza JP (2015). The mistreatment of women during childbirth in health facilities globally: a mixed-methods systematic review. PLoS Med.

[37] Worku AG, Yalew AW, Afework MF (2013). Maternal complications and women’s behavior in seeking care from skilled providers in North Gondar, Ethiopia. PLoS One.

[38] Nwameme AU, Phillips JF, Adongo PB (2014). Compliance with emergency obstetric care referrals among pregnant women in an urban informal settlement of Accra, Ghana. Matern Child Health J.

[39] Combs Thorsen V, Sundby J, Malata A (2012). Piecing together the maternal death puzzle through narratives: the three delays model revisited. PLoS One.

[40] Ibrahim NA, Ajani AWO, Mustafa IA, Balogun RA, Oludara MA, Idowu OE (2017). Road traffic injury in Lagos, Nigeria: assessing Prehospital care. Prehosp Disaster Med.

[41] Medicins Sans Frontières. Nigeria (2018). Saving lives in a three-wheeled ambulance . Featured Videos.

[42] Wilson A, Hillman S, Rosato M, Skelton J, Costello A, Hussein J (2013). A systematic review and thematic synthesis of qualitative studies on maternal emergency transport in low- and middle-income countries. Int J Gynecol Obstet.

[43] Akinwotu E (2015). In Lagos, the traffic jams can add four hours to your commute. Transport.

[44] Panciera R, Khan A, Rizvi SJR, Ahmed S, Ahmed T, Islam R (2016). The influence of travel time on emergency obstetric care seeking behavior in the urban poor of Bangladesh: a GIS study. BMC Pregnancy Childbirth.

[45] Banke-Thomas A, Wright K, Sonoiki O, Ilozumba O, Ajayi B, Okikiolu O (2017). Multi-stakeholder perspectives on access, availability and utilization of emergency obstetric care services in Lagos, Nigeria: a mixed-methods study. J Public Health Africa.

[46] Niyitegeka J, Nshimirimana G, Silverstein A, Odhiambo J, Lin Y, Nkurunziza T (2017). Longer travel time to district hospital worsens neonatal outcomes: a retrospective cross-sectional study of the effect of delays in receiving emergency cesarean section in Rwanda. BMC Pregnancy Childbirth.

[47] Mkoka DA, Goicolea I, Kiwara A, Mwangu M, Hurtig A (2014). Availability of drugs and medical supplies for emergency obstetric care : experience of health facility managers in a rural district of Tanzania. BMC Pregnancy Childbirth.

[48] Daniels AA, Abuosi A (2020). Improving emergency obstetric referral systems in low and middle income countries: a qualitative study in a tertiary health facility in Ghana. BMC Health Serv Res.

[49] Chi PC, Bulage P, Urdal H, Sundby J (2015). Barriers in the delivery of emergency obstetric and neonatal Care in Post-Conflict Africa: qualitative case studies of Burundi and northern Uganda. PLoS One.

[50] Adewole OA, Fadeyibi IO, Kayode MO, Giwa SO, Shoga MO, Adejumo AO (2012). Ambulance services of Lagos state, Nigeria: a six-year (2001-2006) audit. West Afr J Med.

[51] Oni B, Odukoya O, Okunowo A, Ojo O, Abatan Y (2016). A comparative assessment of the awareness of danger signs and practice of birth preparedness and complication readiness among pregnant women attending rural and urban general hospitals in Lagos state. Sahel Med J.

[52] Aduloju OP, Akintayo AA, Aduloju T, Akin-Akintayo OO (2017). Birth preparedness and complication readiness among prenatal attendees in a teaching hospital in south West Nigeria. Int J Gynecol Obstet.

[53] Onono M, Odhiambo GO, Congo O, Waguma LW, Serem T, Owenga MA (2019). Narratives of women using a 24-hour ride-hailing transport system to increase access and utilization of maternal and newborn health Services in Rural Western Kenya: a qualitative study. Am J Trop Med Hyg.

[54] World Health Organization (2018). WHO recommendations Intrapartum care for a positive childbirth experience.

[55] Ezeamalu B (2020). Lagos bans tricycles, motorcycles in 15 local govts. Premium Times.

